# Influenza a Virus Inhibition: Evaluating Computationally Identified Cyproheptadine Through In Vitro Assessment

**DOI:** 10.3390/ijms26135962

**Published:** 2025-06-21

**Authors:** Sanja Glisic, Kristina Stevanovic, Andrej Perdih, Natalya Bukreyeva, Junki Maruyama, Vladimir Perovic, Sergi López-Serrano, Ayub Darji, Draginja Radosevic, Milan Sencanski, Veljko Veljkovic, Bruno Botta, Mattia Mori, Slobodan Paessler

**Affiliations:** 1 Laboratory for Bioinformatics and Computational Chemistry, Institute of Nuclear Sciences VINCA, University of Belgrade, 11000 Belgrade, Serbia; 2 Theory Department, National Institute of Chemistry, 1000 Ljubljana, Slovenia; 3 Faculty of Pharmacy, University of Ljubljana, 1000 Ljubljana, Slovenia; 4 Department of Pathology, University of Texas Medical Branch, Galveston, TX 77555, USAjumaruya@utmb.edu (J.M.); 5 Institute for Human Infections and Immunity, University of Texas Medical Branch, Galveston, TX 77550, USA; 6 Infection Biology Laboratory, Department of Medicine and Life Sciences (MELIS), Universitat Pompeu Fabra, Barcelona Biomedical Research Park (PRBB), 08003 Barcelona, Spain; sergi.lopez@upf.edu; 7 Institut de Recerca en Tecnologies Agroalimentaries (IRTA), Centre de Recerca en Sanitat Animal (CReSA, IRTA-UAB), Campus de la Universitat Autònoma de Barcelona, 08193 Bellaterra, Spain; 8 Biomed Protection, Galveston, TX 77550, USA; 9 Department of Chemistry and Technologies of Drugs, Sapienza University of Roma, 00185 Roma, Italy; 10 Department of Biotechnology, Chemistry and Pharmacy, University of Siena, 53100 Siena, Italy

**Keywords:** antiviral, cyproheptadine, influenza, drug resistance, virtual screening

## Abstract

Influenza is still a chronic global health threat, inducing a sustained search for effective antiviral therapeutics. Computational methods have played a pivotal role in developing small molecule therapeutics. In this study, we applied a combined in silico and in vitro approach to explore the potential anti-influenza activity of cyproheptadine, a clinically used histamine H1 receptor antagonist. Virtual screening based on the average quasivalence number (AQVN) and electron–ion interaction potential (EIIP) descriptors suggests similarities between cyproheptadine and several established anti-influenza agents. The subsequent ligand-based pharmacophore screening of a focused H1 antagonist library was aligned with the bioinformatics prediction, and further experimental in vitro evaluation of cyproheptadine demonstrated its anti-influenza activity. These findings provide proof of concept for cyproheptadine’s in silico-predicted antiviral potential and underscore the value of integrating computational predictions with experimental validation. The results of the current study provide a preliminary proof of concept for the predicted anti-influenza potential based on computational analysis and emphasize the utility of integrating in silico screening with experimental validation in the early stages of drug repurposing efforts.

## 1. Introduction

Influenza remains a major worldwide public health issue that causes 5 million severe cases and 290,000 to 650,000 deaths in annual epidemics [1]. Although seasonal influenza vaccines are widely available, their effectiveness differs. Therefore, a large percentage of the population remains vulnerable to infection [2]. Besides vaccination, anti-viral medications are the primary defense against influenza, including neuraminidase inhibitors (NAIs) and baloxavir (marboxil), a cap-dependent endonuclease inhibitor of the viral polymerase [3]. Although the rate of NAI resistance in presently circulating influenza strains is low, NAIs have a limited therapeutic window, and baloxavir (marboxil) resistance has been reported [4,5,6]. The antiviral favipiravir (T-705) has shown promise in the management of severe infections caused by new or re-emerging influenza A strains in cases where other antiviral medications have failed, but its use is restricted because of its possible adverse effects and restricted approval in some countries [7]. A recent review provided a comprehensive summary of licensed antiviral medications, monoclonal antibodies, and compounds being studied in clinical trials, as well as preclinical agents for combating influenza, also outlining novel anti-influenza therapy strategies, including combination therapies and targeted protein degradation [8]. Because of the limited number of drugs approved and the constraints of current approaches for preventing and treating influenza, particularly in light of the emergence of new strains and the danger of drug resistance, researchers are actively searching for novel and effective influenza treatments.

The emergence of drug-resistant pathogens represents a significant barrier to treating infectious diseases; however, drug repurposing (DR) offers a promising opportunity for rapidly identifying viable therapies [9]. DR is a contemporary and widely embraced strategy in drug discovery that offers a promising route to accelerating the development of new anti-influenza therapies by using the known safety and efficacy profiles of existing drugs; old medications are given new indications through the exploration of novel biochemical pathways and therapeutic targets, simultaneously providing economic benefits and enabling faster regulatory approval [10,11].

Several studies have recently been published on repurposing drugs into direct-acting antivirals against influenza. It has been shown that probenecid, an FDA-approved drug for treating gout and high uric acid levels, inhibits influenza A virus replication in vitro and in vivo, suggesting its potential use as a repurposed direct-acting antiviral [12]. In another study, it was found that lifitegrast, an FDA-approved drug for the treatment of dry eye syndrome, was identified as a promising direct inhibitor of the catalytic site of influenza PA endonuclease in silico and in vitro, highlighting its potential antiviral use [13]. Also, two clinical trials involving influenza viruses using repurposed drugs are currently underway. The first trial (phase 2b/3 clinical trial) is studying clarithromycin, naproxen, and oseltamivir in a triple-drug combination; (2) the second trial is investigating the efficacy of the antiparasitic nitazoxanide against influenza viruses (Phase III) [14]. Nitazoxanide has shown potential for re-use as an influenza treatment in a phase IIb/III clinical trial, in which a shorter duration of symptoms in individuals with acute uncomplicated influenza was found [15]. In another phase II clinical trial, nitazoxanide did not show a therapeutic effect in patients with severe acute influenza, suggesting that the drug’s efficacy depends on the influenza severity and the patient’s demographic category [15].

DR for viral infectious disorders combines the screening of bioactive small molecule collections with computational approaches to identify a chemical, route, or biological activity that could be utilized against the virus of interest [14]. With the rise of big data and advanced computational tools, virtual screening (VS) has become a key systematic approach to accelerating drug repurposing by effectively predicting new therapeutic applications through in silico analysis [16]. VS is the in silico equivalent of high-throughput screening for large compound databases. It plays a crucial role in the drug development process by hugely reducing the time and expenses spent in discovering new medications [17]. Computational methods have been essential in advancing small molecule therapeutics for more than thirty years [18]. Numerous compounds that have advanced to the clinical stage of drug development or received FDA approval were identified and/or re-fined through computational techniques [19]. VS serves as a computational counterpart to the high-throughput screening of extensive compound libraries, with a key role in drug discovery due to its advantage of markedly decreasing the time and expenses associated with identifying a new drug [17]. Numerous predictive computational methods have been developed to uncover drug repositioning possibilities for viral infectious disease treatments facing challenges from drug-resistant pathogens [14]. Applying state-of-the-art approaches to computer simulations of complex biological structures is challenging, with advantages and drawbacks. Nevertheless, combining in silico approaches provides a strong basis for repurposing drugs that merit experimental study [20]. Therefore, we applied a combined in silico approach in this study to identify potential anti-influenza candidates.

The findings of a recent study suggest that two FDA-approved antihistamines, car-binoxamine maleate (CAM) and S-(+)-chlorpheniramine maleate (SCM), exhibit strong anti-influenza properties by inhibiting viral entry. These first-generation H1 receptor antagonists are thought to interfere with the endocytic process, especially by preventing the fusion of the viral envelope with the endosomal membrane, a process mediated by HA. Thus, they could potentially be repurposed as anti-influenza treatments [21].

Previously, we proposed a theoretical criterion for the rapid virtual screening of molecular libraries to identify candidate anti-influenza therapeutics targeting the M2 ion channel, NS1, and hemagglutinin [22,23]. A recent study identified histamine H1 receptor antagonists as influenza inhibitors. Considering this, here, we analyzed and defined the AQVN/EIIP domain of H1 receptor antagonists. Through a combined in silico analysis, we identified cyproheptadine as a potential anti-influenza agent, and its anti-influenza activity was subsequently validated in vitro.

## 2. Results

The VS protocol employed a stepwise approach to select candidate anti-influenza inhibitors. A theoretical criterion for the quick virtual screening of molecular libraries to identify anti-influenza treatment candidates that target the M2 ion channel was previously proposed [22], with a subsequent study proposing criteria for the selection of inhibitors targeting the NS1 protein and hemagglutinin (HA) [23].

**Figure 1:**
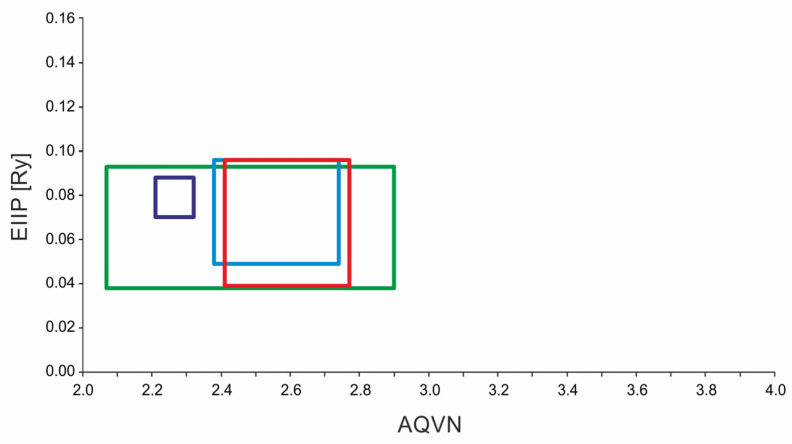
Schematic presentation of the EIIP/AQVN criterion for the selection of candidate anti-influenza compounds through the virtual screening of molecular libraries (green—NS1 inhibitors; blue—HA inhibitors; navy blue—M2 ion channel inhibitors; red—the AQVN/EIIP domain of histamine H1 receptor antagonists).

A recent study identified two histamine H1 receptor antagonists, carbinoxamine and chlorpheniramine, as influenza inhibitors [21]. Building on these findings, we further analyzed the AQVN/EIIP domain of histamine H1 receptor antagonists from the ChEMBL database (https://www.ebi.ac.uk/chembl/explore/target/CHEMBL231 accessed on 30 March 2025) to identify additional candidates for repurposing these drugs against influenza. Our analysis of the histamine H1 receptor antagonists defined an AQVN range of 2.454–2.731 and an EIIP range of 0.052–0.096 (Figure 1), which aligns closely with the HA inhibitor learning set (AQVN: 2.380–2.740; EIIP: 0.049–0.096) and partially overlaps with the NS1 inhibitor learning set (AQVN: 2.069–2.900; EIIP: 0.038–0.093), as previously reported (Figure 1) [23]. Further comparative analysis of the AQVN/EIIP values for carbinoxamine and chlorpheniramine—previously identified as anti-influenza agents [21]—revealed that two HA inhibitors, LI-180299 and BMY-27709, from the previously established learning set [23], exhibited comparable AQVN/EIIP values to carbinoxamine. Expanding this computational screening to other histamine H1 receptor antagonists, we identified six additional compounds with matching AQVN/EIIP characteristics to carbinoxamine: methylpromazine, cyproheptadine, bilastine, phenindamine, carbinoxamine, and fexofenadine. These compounds may have anti-influenza activity, as prior studies have shown that small molecules with similar AQVN and EIIP values interact with common therapeutic targets in various infectious diseases, including HIV, Ebola virus, influenza, malaria, and bacterial infections [23,24,25,26].

**Figure 2:**
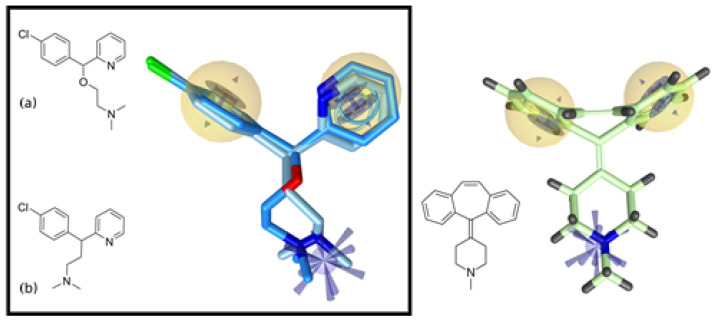
Structures of carbinoxamine (**a**) and chlorpheniramine (**b**) and the alignment of their conformations with the ligand-based pharmacophore. Cyproheptadine as a compound hit in a virtual screening of a library of antihistaminic compounds. Yellow spheres are hydrophobic features; blue spikes shows the positive ionizable area, and blue circles are aromatic ring features.

Subsequently, we performed a ligand-based pharmacophore screening of a focused library of H1 receptor antagonists to identify novel potential anti-influenza compounds based on the previously observed anti-influenza properties of carbinoxamine and chlorpheniramine. A ligand-based pharmacophore was derived from these compounds, and the initial pharmacophore model was reduced to increase the coverage of the chemical space of potential hits fitting the pharmacophore model. The final model comprised two hydrophobic interaction spheres and aromatic rings at approximately the same position to model the importance of the phenyl moieties in both compounds for biological activity. The pharmacophore also contained a positive ionizable region to model the ionic interactions of the amine group within the ethanolamine moiety (blue spikes) (Figure 2).

The discriminatory ability of the pharmacophore model [27] was confirmed through screening 100 decoy molecules generated from the two active molecules using the DUD-E server [28]. The decoy molecules differ in their chemical structure but have similar physical properties, as shown by the highest Tanimoto threshold between the MACCS fingerprints of the drug and the decoy molecule [28]. No decoy molecules were recognized as potential hits; the pharmacophore model was able to effectively identify both compounds in their correct orientation.

To expand the possible chemical space of antihistamines for this new pharmacological indication, we again turned to the ChEMBL database [29] and extracted antihistamines that act on the H1 receptor. A total of 62 compounds formed a focused library, which was screened against the derived pharmacophore model, resulting in 24 hit compounds. One of the hit compounds, cyproheptadine, aligned to the screening pharmacophore, is shown in Figure 2. This compound, also predicted as a hit in the previous bioinformatics-based screening, attracted our particular attention as it can be considered a more rigid structural alternative to the active compounds while also not being significantly structurally different from them. Two phenyl rings present in carbinoxamine and chlorpheniramine are connected in cyproheptadine via the third sevenmembered cycloheptene ring, and the linker ethoxy moiety is replaced by a cyclohexyl moiety, allowing the same distance to be maintained between the core features of the pharmacophore.

The combined results of the AQVN/EIIP analysis and ligand-based pharmacophore screening thus point to cyproheptadine as a possible hit compound among the selected H1 antihistamines. Its identification with both screening models provided us with the confidence to select this compound and conduct an in vitro experimental validation of its antiviral efficacy.

### In Vitro Efficacy of Cyproheptadine Against H1N1 Influenza A Viruses

**Figure 3:**
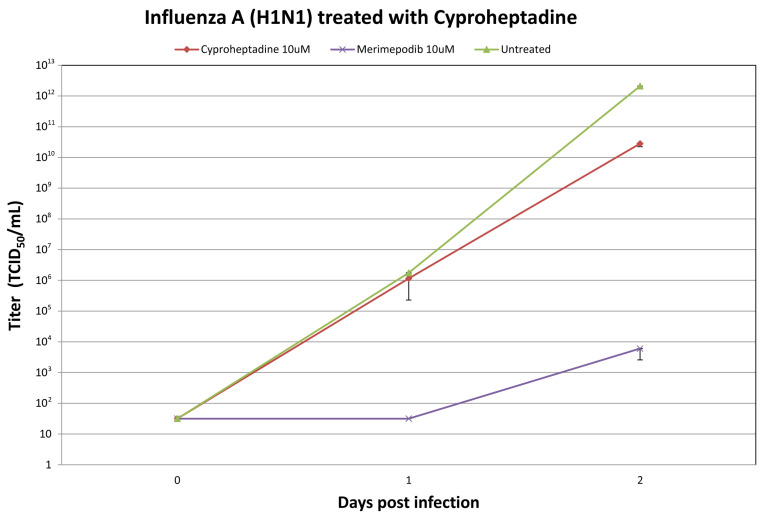
Influenza A/CA/07/2009 (H1N1) viral titers at 0, 1, and 2 days post-infection (dpi) after treatment with cyproheptadine with the indicated drug concentrations. Ten micromolar (10 μM) merimepodib was used as a positive control. The results are plotted as the means of triplicate observations, with standard deviations shown.

Cyproheptadine was administered to cells infected with H1N1 influenza A viruses, resulting in a reduction in the production of infectious viruses. Treatment with 10 μM cyproheptadine led to substantial decreases in the H1N1 virus titers at +1 and +2 days post-infection (Figure 3). The 10 μM cyproheptadine treatment resulted in viral titer reductions at +2 days post-infection. Based on the TCID_50_ assay data, this corresponds to a decrease of nearly two orders of magnitude compared to untreated controls. In our in vitro assay, cyproheptadine (BBN9, 10 µM) reduced the viral titers by approximately 74-fold at 48 h post-infection compared to the untreated controls, based on the TCID_50_ measurements. As a positive treatment control, the influenza A virus was pre-mixed with 10 µM of merimepodib, an IMPDH inhibitor with known antiviral activity against various viruses, including influenza A virus [30,31]. Cyproheptadine reduced influenza virus production in the experiments with influenza A/CA/07/2009 (H1N1) (Figure 3).

## 3. Discussion

The existing strategies for preventing and treating influenza A and B infections are inadequate, primarily because of the increasing clinical application of approved antivirals, resulting in the development of resistant viral strains [32]. The quest for innovative preventive and therapeutic strategies to diminish drug resistance and the risks of pandemic virus outbreaks faces significant challenges due to the costly, lengthy, and considerably risky process of drug development. Therefore, repurposing current drugs offers a hopeful approach to treating viral infections, including those of influenza A and B. Abundant computational methods have been developed to discover candidate drugs to be repurposed for targeting influenza viruses [33]. Previous research has demonstrated that the EIIP/AQVN criterion serves as an effective filter for virtually screening molecular libraries designed to identify potential inhibitors of viral infections, such as HIV and Ebola virus [26,34,35]. By using this approach, Ibuprofen was proposed as a potential inhibitor of the Ebola virus, which was subsequently confirmed through experimental validation [36,37]. This approach has also been applied in the search for new influenza virus treatments. The EIIP/AQVN criterion is unique in virtual screening because, among over 3300 molecular descriptors currently used to characterize organic molecules, only the EIIP and AQVN descriptors capture molecules’ long-range electronic properties [38]. These properties are essential for understanding and predicting molecular recognition and interaction at distances greater than typical short-range chemical bonding. Therefore, the EIIP/AQVN criterion is uniquely suited for identifying bioactive compounds—such as anti-influenza agents—through virtual screening, where long-range interactions often play a critical role in the initial recognition between small molecules and biological targets. The results of the in silico screening using the EIIP/AQVN filter, a ligand-based virtual screening, and molecular docking led to the identification of several candidate compounds that may represent promising dual inhibitors of both wild-type and adamantane-resistant influenza A virus strains. Guanethidine, as the top-ranked compound in silico, was later tested in vitro, demonstrating measurable anti-influenza activity in cell culture [22]. Similarly, the anticholinergic agent cycrimine has been reported as a potential therapeutic candidate for combating the influenza A virus, demonstrating antiviral efficacy across various subtypes in vitro [39]. The EIIP/AQVN criterion was applied to the ZINC Natural Product database in another study, followed by ligand-based screening and molecular docking, to identify a possible NS1 inhibitor, a natural compound that was experimentally tested and found to inhibit viral replication in cell culture [23].

This study presents a comprehensive virtual screening approach integrating AQVN/EIIP-based filtering and ligand-based pharmacophore modeling to identify histamine H1 receptor antagonists with potential anti-influenza activity, simultaneously building on previously established criteria for screening influenza inhibitors targeting viral proteins such as M2, NS1, and hemagglutinin (HA) [22,23]. Previous research has identified carbinoxamine and chlorpheniramine—both first-generation H1 receptor antagonists—as compounds capable of inhibiting influenza virus by interfering with the endocytic process, particularly the HA-mediated fusion of viral and endosomal membranes [21]. The overlap of the AQVN/EIIP profiles across these different molecule classes suggests that they could share a drug target. This finding aligns with earlier work demonstrating that small molecules with similar AQVN/EIIP values can act on common therapeutic targets across various pathogens, including HIV, Ebola virus, malaria, and bacterial infections [23,24,25,26].

Using the AQVN/EIIP criteria, we screened histamine H1 receptor antagonists. We observed a substantial overlap between the H1 receptor antagonists’ AQVN/EIIP domains and the AQVN/EIIP domains of HA inhibitors, and partial overlap with the AQVN/EIIP domains of NS1 inhibitors [23]. Matching AQVN/EIIP characteristics were also observed between reported HA fusion inhibitors, such as BMY-27709 and LY-180299 [23], and carbinoxamine, previously identified as anti-influenza agents [21]. HA fusion inhibitors have been shown to inhibit viral entry by stabilizing HA in its neutral conformation, thus preventing the low-pH-induced fusogenic transition required for viral fusion and genome release [40,41,42]. Such inhibitors act at a late stage of endocytosis by blocking membrane fusion, a mechanism distinct from those affecting viral attachment or neuraminidase-mediated release. By extending this computational analysis to other histamine H1 receptor antagonists, we identified six additional compounds—including cyproheptadine—that share AQVN/EIIP characteristics with carbinoxamine, which was previously reported to have anti-influenza activity. In addition to its overlap with HA inhibitors, cyproheptadine is in the AQVN/EIIP domain with previously identified NS1 inhibitors. This finding could indicate that cyproheptadine potentially exhibits multi-target activity that may extend from viral entry to intracellular replication. Such dual or multi-target mechanisms are also of particular importance in the context of antiviral drug development, as they may reduce the likelihood of resistance, as recently demonstrated in a study by Lao and coworkers [43], in which novel compounds capable of simultaneously targeting hemagglutinin and neuraminidase demonstrated the therapeutic advantage of multi-target engagement, preventing the emergence of resistant influenza strains.

To complement the AQVN/EIIP screening, we also applied ligand-based pharmacophore modeling using the structural features of the histamine H1 receptor antagonists carbinoxamine and chlorpheniramine, which demonstrated antiviral properties against influenza. The pharmacophore model, as a result, captured key features such as hydrophobicity, aromatic ring positions, and a positively ionizable region, which is crucial for biological activity. Validation through a decoy screening using the DUD-E database demonstrated specificity and no false positives, indicating a strong predictive power.

We selected cyproheptadine for further investigation as it was reported as a hit in both the AQVN/EIIP domain analysis and the ligand-based pharmacophore screening. This convergence across two independent computational approaches strengthens the rationale for its selection, particularly given its shared physicochemical characteristics with other H1 antihistamines previously shown to exhibit anti-influenza activity [21]. Such an integrative in silico strategy—despite the inherent limitations in modeling biological complexity—provides a robust framework for generating repurposing hypotheses that merit experimental validation [20], as confirmed by the in vitro results obtained in this study.

Cyproheptadine was singled out among the other candidates due to it exhibiting essential pharmacophoric features while presenting a more rigid molecular structure, which may augment its binding stability and specificity. Furthermore, its previously reported antiviral activity against the African swine fever virus and hepatitis C virus [44,45] suggest its potential as a broad-spectrum antiviral agent.

The development of new antiviral agents is still costly and time-intensive, representing a significant hindrance in swiftly responding to viral threats, including emerging resistance and potential pandemics. Drug repurposing has arisen as a practical and efficient strategy, particularly for influenza A and B. Approved drugs are especially valued in this approach, as they offer known safety profiles and established pharmacokinetics. Particularly, analyses of newly marketed drugs have shown that a substantial portion is derived from previously approved agents or clinical candidates, emphasizing the strategic role of repurposing in modern drug discovery [46].

Cyproheptadine is a first-generation H1 antihistamine that was developed in the 1960s. It is widely used for allergic conditions and is approved in several countries, including the United States, for its orexigenic (i.e., appetite-stimulating) properties [47]. Its wide clinical use, as well as its favorable tolerability profile and wide availability, including over-the-counter availability in certain countries, make it a promising candidate for drug repurposing. Also, data from the French national pharmacovigilance database and the relevant literature indicate that cyproheptadine is generally well tolerated. Adverse effects of cyproheptadine have been reported as mild and reversible neurological symptoms, such as drowsiness and dizziness [48]. Furthermore, hepatic complications are rare and generally resolve after discontinuation of the drug. Considering the favorable safety profile of cyproheptadine, its availability and affordability make it a promising candidate for repurposing as an anti-influenza drug. The findings from our current study, based on computational screening and in vitro validation, suggest cyproheptadines’s potential anti-influenza activity. The results of the current study and the well-known clinical characteristics of the drug warrant further investigation of cyproheptadine in influenza, especially given the urgent need for safe and cost-effective anti-influenza treatments.

Direct-acting antivirals (DAAs) are the preferred therapeutic approach for influenza, as host-directed therapies, regardless of their potential for broad-spectrum activity and resistance avoidance, have not yet reached clinical use due to safety barriers and the risk of off-target effects [48,49]. In this context, the identification of cyproheptadine as a potential DAA supports its possible utility as a preliminary model compound in influenza-related drug repurposing studies.

In several prior studies, it was demonstrated that cyproheptadine exhibits direct antiviral activity. In vitro results from the study indicate that it inhibited the proliferation of African swine fever virus (ASFV) by targeting the viral D1133L gene, leading to a reduction in its transcription and protein expression while displaying low cytotoxicity [44]. Additionally, in other research studies, cyproheptadine demonstrated anti-HCV activity, likely through interference with endosomal processes required for viral entry and possibly by interfering with other stages of the viral life cycle [45]. Also, it is important to note that a similar mechanism—impairment of endocytosis—has been proposed for other first-generation antihistamines, including carbinoxamine maleate (CAM) and S-(+)-chlorpheniramine maleate (SCM), which exhibit potent antiviral effects against various influenza A virus strains in both in vitro and in vivo models. In a previous research study, it was reported that CAM and SCM likely act by blocking viral entry at an early stage of replication, not affecting hemagglutinin-mediated binding or neuraminidase-mediated release. Since cyproheptadine shares structural and pharmacological characteristics with these compounds, it can be hypothesized that it may act through a similar mechanism by interfering with endocytic pathways required for viral entry.

Cyproheptadine demonstrated measurable antiviral activity against influenza A virus in vitro following its identification through in silico screening. While the direct translation of in vitro results to clinically achievable plasma concentrations has not been fully determined, pharmacokinetic studies report that the standard oral dosing of cyproheptadine (up to 16 mg/day) results in peak plasma concentrations around 0.1–0.14 μM [50,51]. Nevertheless, cyproheptadine is a widely used and well-tolerated drug and its favorable safety profile, affordability, and accessibility support further exploration of its antiviral potential. Moreover, its tendency to possibly accumulate in target tissues, such as the lungs, and the application of alternative delivery strategies or combination therapies could enhance its effectiveness against influenza infection.

## 4. Materials and Methods

### 4.1. Dataset Preparation

To establish a predictive model for identifying potential influenza inhibitors, we first compiled a set of active compounds targeting the histamine H1 receptor. These data were obtained from the ChEMBL Target Report Card (CHEMBL613740; https://www.ebi.ac.uk/chembl/target/inspect/CHEMBL613740 accessed on 30 March 2025), (Table S1). A reference dataset of known influenza HA inhibitors, NS1 inhibitors, and M2 ion channel inhibitors previously defined in earlier studies [22,23] was used for comparative analysis.

### 4.2. AQVN/EIIP-Based Virtual Screening

For each compound in the histamine H1 receptor antagonist dataset from the ChEMBL Target Report Card (CHEMBL613740; https://www.ebi.ac.uk/chembl/target/inspect/CHEMBL613740, accessed on 30 March 2025), we calculated two molecular descriptors: the average quasivalence number (AQVN) and the electron–ion interaction potential (EIIP). Table S1 presents the AQVN/EIIP values calculated for histamine H1 receptor antagonists. The two H1 antagonists—carbinoxamine and chlorpheniramine—previously identified as potential influenza inhibitors [21], were selected as reference compounds. Their AQVN/EIIP values were computed and served as the baseline for further comparison. A focused library of H1 receptor antagonists was screened to identify additional compounds with AQVN/EIIP values similar to H1 antagonists showing anti-influenza activity.

#### EIIP/AQVN Values

The EIIP for organic molecules can be determined using the following simple equation derived from the “general model pseudopotential” [25]:

EIIP = 0.25 Z* sin(1.04 π Z*)/2π(1)

where Z* is the average quasi valence number (AQVN), determined by

Z* = ∑_i=1,m_(n_i_ Z_i_/N) (2)

where Z_i_ is the valence number of the ith atomic component, n_i_ is the number of atoms of the ith component, m is the number of atomic components in the molecule, and N is the total number of atoms. EIIP values, calculated according to Equations (1) and (2), are expressed in Rydberg units (Ry).

### 4.3. Pharmacophore Modeling and Screening

The conformations of carbinoxamine and chlorpheniramine ligands were minimized using the MMFF94 force field. The 3D conformer generator in LigandScout [52] was used to generate the multiconformational models required for pharmacophore modeling with Best settings: up to 200 output conformers per molecule, RMS threshold 0.8 Å for duplicate conformers, and up to 4000 intermediate conformers per molecule. A total of 30 different conformations were generated for carbinoxamine and 24 for chlorpheniramine. By dynamically aligning [53] the conformers in the LigandScout ligand-based module, 10 ligand-based pharmacophore models were generated. The models were evaluated using a scoring algorithm that considered pharmacophore alignment and atomic shape overlap. The model with the highest score of 0.9627 was selected (Figure S1) and visual inspection revealed that other generated pharmacophores were highly similar to this model. The number of pharmacophore features in the model was reduced to increase the coverage of the chemical space of hits that can fit the pharmacophore model while still ensuring that the molecular recognition pattern of the active compounds was sufficient (Figure 2) [27].

To check the discriminatory ability of the developed pharmacophore model, SMILES of both active compounds were entered as template molecules into the Database of Useful Decoys: Enhanced (DUD-E Server) [28] to create 100 decoys: 50 for each ligand. The decoys were then screened against the pharmacophore model in the virtual screening module. The ChEMBL database [29] served to assemble a focused library of 62 compounds that act as H1-receptor antagonists. These were converted into a multifunctional format with Fast settings: an RMS threshold of 0.8 Å for duplicate conformers, limit of 30,000 conformers generated per molecule, maximum of 4000 intermediate conformers per molecule, and a maximum 25 output conformers per molecule. The library was then screened against the pharmacophore model and the alignment of hit molecules was assessed using the Pharmacophore Fit scoring function. Only compounds that aligned with all pharmacophore features were considered hits.

### 4.4. In Vitro Efficacy Testing Against H1N1 Influenza A Viruses

The experiment began by pre-mixing influenza A/CA/07/2009 (H1N1) virus with cyproheptadine, followed by an hour-long incubation at 37 °C. Madin–Darby canine kidney (MDCK) cells in 12-well plates, reaching 85–95% confluency, were washed twice with serum-free media before being infected with the virus/drug mixtures. Each treatment group was tested in triplicate.

As a positive treatment control, the influenza A virus was pre-mixed with 10 µM of merimepodib, an IMPDH inhibitor with known antiviral activity against various viruses, including influenza [30,31].

After an hour of incubation at 37 °C with 5% CO_2_, cells were washed once with se-rum-free media, and the appropriate concentration of test drug was added to each well. Negative control wells were mock-infected, while virus control wells were infected but left untreated. The cells were then kept at 37 °C with 5% CO_2_, and samples were collected at 0, 24, and 48 h post-infection, then stored at −80 °C until analysis.

In order to measure the reduction in the production of infectious progeny, each sample was diluted at a 1:10 ratio and used to inoculate cells in 96-well plates with approximately 85–95% confluency to determine viral titers using a 50% tissue culture infective dose (TCID_50_) assay. Growth curves for each virus were constructed based on individual titers collected at the specified time points.

## 5. Conclusions

In this study, we present a theoretical and preliminary experimental framework for identifying potential influenza inhibitors using a combined computational and in vitro approach. Cyproheptadine was identified as a compound of interest, showing measurable antiviral activity against influenza A virus in cell-based assays. Further studies are needed to investigate cyproheptadine’s activity against other influenza A subtypes and to evaluate its performance in relevant in vivo systems. Future work may also aim to elucidate cyproheptadine’s precise mechanism of action.

The findings of the present study provide a preliminary proof of concept for the anti-influenza potential of cyproheptadine and underscore the value of integrating computational predictions with experimental validation during the early stages of drug re-purposing research. These results further reinforce the importance of combining in silico and experimental approaches in the identification of repurposing candidates.

## Data Availability

The original contributions presented in the study are included in the article/Supplementary Materials, further inquiries can be directed to the corresponding author.

## Supplementary Materials

The supporting information can be downloaded at https://www.mdpi.com/article/10.3390/ijms26135962/s1.
