# Supplementary material for: Influenza a Virus Inhibition: Evaluating Computationally Identified Cyproheptadine Through In Vitro Assessment
Source: Int J Mol Sci. 2025 Jun 21;26(13):5962. doi: 10.3390/ijms26135962 (PMC12249625; doi:10.3390/ijms26135962)
Supplement: Supplementary file 1 [file ijms-26-05962-s001.zip › Figure S1.pdf]

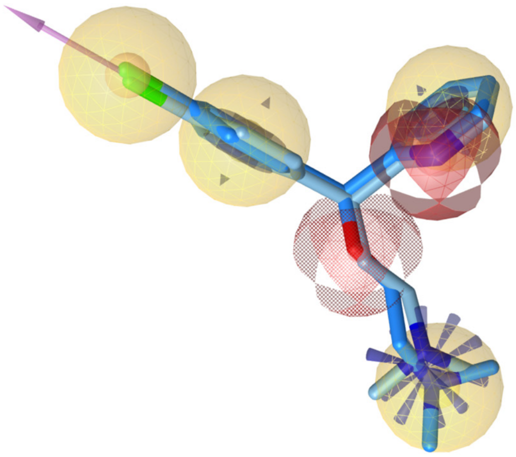

Figure S1: Active compounds carbinoxamine and chlorpheniramine, aligned on the de-rived full ligand-based pharmacophore model. Red spheres show hydrogen bond acceptors, yellow spheres show hydrophobic features, pink arrows show halogen bond donors, blue arrows show positive ionizable areas, and blue circles show aromatic ring features
